# Phenolic Profile and Cholinesterase Inhibitory Properties of Three Chilean Altiplano Plants: *Clinopodium gilliesii* (Benth.) Kuntze [Lamiaceae], *Mutisia acuminata* Ruiz & Pav. var. hirsuta (Meyen) Cabrera, and *Tagetes multiflora* (Kunth) [Asteraceae]

**DOI:** 10.3390/plants12040819

**Published:** 2023-02-12

**Authors:** Carlos Fernández-Galleguillos, Felipe Jiménez-Aspee, Daniel Mieres-Castro, Yeray A. Rodríguez-Núñez, Margarita Gutiérrez, Luis Guzmán, Javier Echeverría, Claudia Sandoval-Yañez, Oscar Forero-Doria

**Affiliations:** 1 Departamento Biomédico, Facultad de Ciencias de la Salud, Universidad de Antofagasta, Antofagasta 1270300, Chile; 2 Institute of Nutritional Sciences, Department of Food Biofunctionality (140b), Garbenstr. 28, 70599 Stuttgart, Germany; 3 Institute of Biological Sciences, University of Talca, 1 Poniente 1141, Talca 3465548, Chile; 4 Departamento de Química, Facultad de Ciencias Exactas, Universidad Andrés Bello, Republica 275, Santiago 8370146, Chile; 5 Laboratorio de Síntesis y Actividad Biológica, Instituto de Química de Recursos Naturales, Universidad de Talca, 1 Poniente No. 1141, Talca 3460000, Chile; 6 Departamento de Bioquímica Clínica e Inmunohematología, Facultad de Ciencias de la Salud, Universidad de Talca, Talca 3460000, Chile; 7 Departamento de Ciencias del Ambiente, Facultad de Química y Biología, Universidad de Santiago de Chile, Santiago 9170022, Chile; 8 Grupo de Investigación Química y Bioquímica Aplicada a la Biotecnología, Instituto de Ciencias Químicas Aplicadas, Facultad de Ingeniería, Universidad Autónoma de Chile, Av. Pedro de Valdivia 2541, Santiago 8320000, Chile; 9 Departamento de Ciencias Básicas, Facultad de Ciencias, Universidad Santo Tomás, Talca 3460000, Chile

**Keywords:** phenolic profile, HPLC-DAD, *Clinopodium gilliesii*, *Mutisia acuminata*, *Tagetes multiflora*, cholinesterase inhibition

## Abstract

This research aimed to identify the phenolic profile and composition of the aerial parts of three native species used in traditional medicine in the Andean Altiplano of northern Chile: *Clinopodium gilliesii* (Benth.) Kuntze [Lamiaceae] (commonly known as Muña-Muña), *Mutisia acuminata* Ruiz & Pav. var. hirsuta (Meyen) Cabrera [Asteraceae] (commonly known as Chinchircoma), and *Tagetes multiflora* (Kunth), [Asteraceae] (commonly known as Gracilis), as well as to evaluate their potential inhibitory effects against acetylcholinesterase (AChE) and butyrylcholinesterase (BChE). Polyphenolic enriched-extracts (PEEs) of the species were prepared and analyzed and the main components were quantified using HPLC-DAD. In total, 30 phenolic compounds were identified and quantified in all species, including simple phenolics, hydroxycinnamic acids, flavan-3-ols (monomers and polymers), flavanones, and flavonols. In addition, other main phenolics from the extracts were tentatively identified by ESI-MS-MS high-resolution analysis. *T. multiflora* extract showed the greatest anti-AChE and BChE activity in comparison with *C. gilliesii* and *M. acuminata* extracts, being the anti-AChE and BChE activity weak in all extracts in comparison to galantamine control. To comprise to better understand the interactions between cholinesterase enzymes and the main phenolics identified in *T. multiflora*, molecular docking analysis was conducted.

## 1. Introduction

The Andean Altiplano is a highland plateau region (above 3700 m.a.s.l.) located in west-central South America, in the geographical limit between northwest Argentina, southern Peru, western Bolivia, and northern Chile. This region is characterized by extreme temperatures, high radiation, irregular precipitation, hypersaline soils, and scarce nutrient availability. Plants growing in the Andean Altiplano are well known for having great natural sources of phytochemical compounds that are present in high concentrations in response to extreme environmental conditions [1]. Chilean natives have used Altiplano plants as part of their traditional medicine to treat a wide range of illnesses and ailments [2,3,4]. In this sense, native Andean Altiplano medicinal plants represent an underexplored valuable source of bioactive compounds with potential pharmacological applications [2,3,4]. Among the medicinal Altiplano plants, members of the Lamiaceae and Asteraceae families have been thoroughly investigated due to their traditional uses in cooking, as aromatic plants, as well as in traditional medicine [2,3,4]. In the scientific literature, there are several reports on their health-beneficial properties, including their antioxidant [5], anti-inflammatory [6], antibacterial [7], antifungal [8], and antiprotozoal activities [9], among many others.

*Clinopodium gilliesii* Kuntze [Lamiaceae], *Mutisia acuminata* var. *hirsuta* (Meyen) Cabrera [Asteraceae], and *Tagetes multiflora* Kunth [Asteraceae) are three native species that grow in the Andean Altiplano in the north of Chile [10,11,12]. *C. gilliesii*—commonly known as Muña-Muña—is a highly valued and aromatic native herb that grows between Arica y Parinacota and Atacama regions and has been used in traditional medicine to treat high blood pressure, stomachaches, heart pain, female sterility, and as an aphrodisiac [10]. *M. acuminata*—commonly known as Chinchircoma—is a native small shrub distributed in the Arica y Parinacota region used in traditional medicine for the treatment of gastric ulcers and respiratory problems [11,13]. *T. multiflora*—commonly known as Gracilis—can also be found growing between the Arica y Parinacota and the Atacama region. This plant is traditionally used for stomachache, and abdominal distension, and is mixed with *Ephedra americana* Humb. & Bonpl. ex Willd. [Ephedraceae] for the treatment of urinary tract infection [12]. Several monoterpenes have been identified from the essential oils of the aerial parts from Chilean [10] and Argentinian *C. gilliessi* [14]. In addition, hydroxycinnamic acid derivatives and flavonoids have been reported in polar extracts from *C. gilliessi* (previously called *Satureja parvifolia* Phil.) [15]. The antimicrobial [1], antiplasmodial [16], antiprotozoal, and cytotoxic properties were reported for this species [17]. In addition, the antioxidant and cholinesterase inhibitory activity from Argentinian *C. gilliese* have also been investigated [15]. Regarding *M. acuminata*, different compounds have been identified including arbutin, lupeol and pseudotaraxasterol, β-amyrin, quercetin, quercetin-3-*O*-glucuronide, and isorhamnetin-3-*O*-glucuronide [11,13]. In addition, *M. acuminata* showed antimicrobial activity against *Listeria monocytogenes*, *Staphylococcus aureus*, and *Pseudomonas aeruginosa* [18]. From the aerial parts of *T. multiflora*, only the chemical composition of the essential oils has been investigated, being acyclic monoterpenoids the major components identified [19]. To the best of our knowledge, there are no reports regarding the phenolic profile of these species growing in the Chilean Altiplano.

Compounds that show different and specific enzyme inhibitory capacities have great significance in the management of several diseases [20]. The inhibition of key enzymes linked to Alzheimer’s disease (AD), such as acetylcholinesterase (AChE) and butyrylcholinesterase (BChE), is an important task for identifying novel and safe medicine from natural sources, especially those found in native plants [20,21]. The inhibition of AChE improved the levels of acetylcholine (ACh) in the postsynaptic membrane, reducing cognitive deterioration in patients with AD [21]. Nowadays, the approved drugs for the treatment of AD are cholinesterase inhibitors. However, they are characterized by adverse effects such as hepatotoxicity and gastrointestinal problems [22]. Compared to synthetic drugs, bioactive compounds from natural sources—including polyphenolic antioxidant compounds from Altiplano plants—may have promising effects [23]. However, there are few studies evaluating the potential inhibitory properties against AChE and BChE [21,24].

Thus, the objective of this research was to identify and quantify the phenolic compounds present in three Chilean Altiplano plants and to evaluate their potential inhibitory effects against cholinesterases. For this purpose, the polyphenolic-enriched extracts (PEEs) from the aerial parts of *C. gilliesii*, *M. acuminata*, and *T. multiflora* were investigated using HPLC-DAD and assessed in vitro as cholinesterase inhibitors against AChE and BChE enzymes. Main compounds were studied by molecular docking to investigate intermolecular interactions with AChE and BChE.

## 2. Results and Discussion

Aerial parts of *C. gilliesii*, *M. acuminata*, and *T. multiflora* were used as the botanical source of polyphenols, which were identified and quantified by HPLC-DAD and ESI-MS-MS high-resolution analysis and investigated as potential cholinesterase inhibitors.

### 2.1. Extraction Yield

The *w/w* percent yield of the PEE from 1.0 g of EtOH extract was 24.93, 32.81, and 26.97% *w/w* for *C. gilliesii*, *M. acuminata*, and *T. multiflora*, respectively. The yield results obtained were similar to other previous studies related to these north-Andean species [15,25,26].

### 2.2. Tentative Identification and Quantification of Compounds

The HPLC-DAD analysis of the PEEs from *C. gilliesii*, *M. acuminata*, and *T. multiflora* allowed the identification and quantification of 30 phenolic compounds by matching with commercial standards (Table 1). In addition, other main compounds from *M. acuminata* and *T. multiflora* were tentatively identified by high-resolution mass spectrometry analysis (Figure S1 and S2). Some phenolic compounds were identified and reported in this study for the first time for these species. A detailed analysis of each species is provided below.

#### 2.2.1. *Clinopodium gilliesii* Kuntze [Lamiaceae]

**Figure 1:**
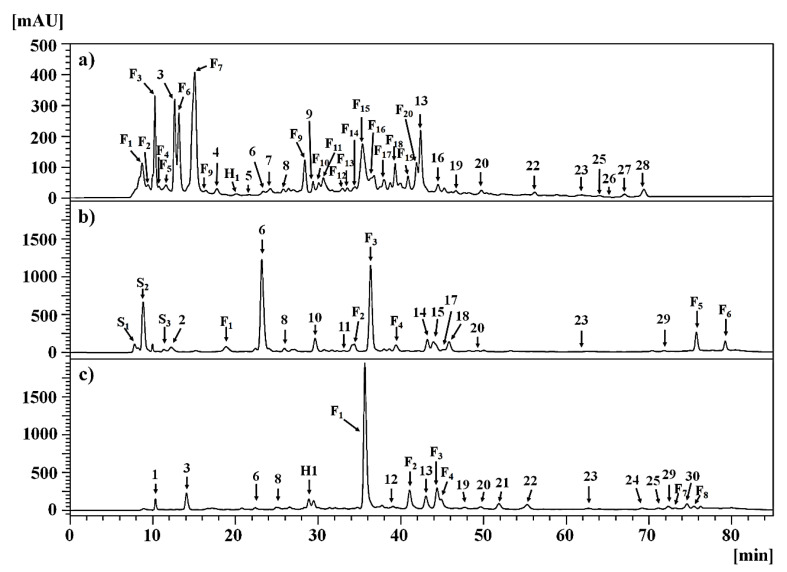
HPLC-DAD chromatogram of the PEEs measured at 280 nm from the aerial parts of (**a**) *Clinopodium gilliesii*, (**b**) *Mutisia acuminata*, and (**c**) *Tagetes multiflora*. F: flavonoid-type, S: simple phenolic; H: hydroxycinnamic acid-type. The number refers to compounds identified by matching with commercial standards.

The HPLC-DAD chromatogram of the PEEs of *C. gilliesii* is shown in Figure 1a and the total content of compounds is shown in Table 1. Two main compounds were detected as simple phenolic derivatives, vanillic acid and *p*-hydroxybenzoic acid, compounds **3** and **4.** The compounds were identified by matching with commercial standards such as vanillic acid and *p*-hydroxybenzoic acid (*p*-HBA), respectively. The total content of simple phenols derivatives was 13.80 ± 0.45 mg of *p*-HBA equivalents/g PEE. Vanillic acid was the major phenolic with 12.37 mg of *p*-HBA equivalents/g PEE. Both phenolic acids have been described in species belonging to the Lamiaceae family and with high content in the genus *Clinopodium* [27].

Compounds **6** and **8** presented the same retention time and UV profile of the commercial standards and were identified as 3-*O*-caffeoylquinic acid (3CQA) and *trans*-caffeic acid, respectively. Compound H_1_ was tentatively identified as a coumaroylquinic acid derivative due to its strong absorption of band I at 323 nm and the UV spectrum characteristic of coumaroylquinic acids [28]. The total content of hydroxycinnamic acids (HCA) was 4.27 ± 0.35 mg of 3CQA equivalents/g PEE. Cabana et al. (2013) [15] reported the presence of caffeoylquinic acid derivatives, including 3CQA, in agreement with our results.

Flavonoids such as flavan-3-ols, flavanones, and flavonols were the main compounds in the PEE. Compounds **5** and **7** were identified by matching with standards as (+)-catechin (CAT) and B-type procyanidin dimer, respectively. Compounds **F_1_**, **F_5_**, **F_8_**, **F_10_**, **F_12_, F_16,_** and **F_17_** were identified as flavan-3-ol derivatives following the UV profile of band II, with λ_max_ at 280 nm and spectra similar to that of catechin [29]. The total content of flavan-3-ol derivatives was 70.00 mg of CAT equivalents/g PEE. This high content of flavan-3-ol derivatives was also reported in other studies with this species, reporting high content of epigallocatechin-3-*O*-gallate and epicatechin-3-*O*-gallate [15]. Compounds naringin (**19**) and naringenin (NAR) (**26**) were identified in this extract by matching with commercial standards. Compounds **F_2_**, **F_3_**, **F_4_**, **F_6_**, **F_7_**, **F_9_**, **F_11_**, **F_13_**, and **F_14_** were tentatively identified as flavanones. Compounds **F_2_**, **F_3_**, **F_4,_** and **F_6_** presented a λ_max_ at 286–289 with a characteristic shoulder at 330 nm in agreement with the UV spectra reported for eriodictyol [30] and previous reports with this species [15]. Compounds **F_7_**, **F_9_**, **F_11_**, **F_13,_** and **F_14_** presented a λ_max_ at 283–285 nm with a characteristic shoulder at 330 nm and were tentatively identified as naringenin derivatives following the UV spectra [30]. The lower retention time suggests that these peaks could present extra glycosyl units. The flavanone derivatives were the main compounds in the PEE with a total content of 93.08 mg of NAR equivalents/g PEE. Similar results were found in other studies with polar extracts of *C. gilliesii* reporting high content of flavonoids with a predominant presence of naringenin and eriodictyol [15,17,31]. Compounds **9**, **13**, and **28** were identified by matching with standards such as luteolin-7-*O*-glucuronide, luteolin-7-*O*-glucoside, and luteolin, respectively. Compounds **F_15_**, **F_18_**, **F_19,_** and **F_20_** presented similar UV spectra to Luteolin derivatives reported in the literature [15,30], with strong absorption of the band I between 342 and 369 nm and with a shoulder at 265 nm being identified as luteolin derivatives. Compounds **16**, **20**, **22**, **23**, **25**, and **27** were identified by matching with standards such as quercetin-3-*O*-galactoside (**16**), quercetin-3-*O*-glucoside (Q3G) (**20**), kaempferol-3-*O*-glucoside (**22**), isorhamnetin-3-*O*-rutinoside (**23**), isorhamnetin-3-*O*-glucoside (**25**), and apigenin-7-*O*-glucoside (**27**). The main flavonols in the extract were luteolin and quercetin derivatives and the total content was 48.39 mg of Q3G equivalents/g PEE. Previous studies reported high content of luteolin derivatives in polar extracts of this species [15,17,31].

#### 2.2.2. *Mutisia acuminata* Ruiz & Pav. var. hirsuta (Meyen) Cabrera [Asteraceae]

The HPLC-DAD chromatogram of the PEE of *M. acuminata* is shown in Figure 1b and the total content of compounds is shown in Table 1. Compound **10** was identified by matching with the standard salicylic acid. The compounds **S_1_**, **S_2_**, and **S_3_** were identified as simple phenols derivatives. The compounds **S_1_**, **S_2_**, and **S_3_** presented UV spectra similar to arbutin derivatives according to the literature [32,33]. The QTOF analysis of the PEE of *M. acuminata* showed the presence of the aglycone arbutin (hydroquinone) (Figure S1); however, the identity remains to be confirmed. The total content of simple phenols derivatives was 66.99 mg of *p*-HBA equivalents/g PEE. Compounds **S_1_**, **S_2_**, and **S_3_** were the major simple phenols in the extract. Previous studies with extracts of *M. acuminata* also reported a high content of arbutin [18,32]

Compounds **2**, **6**, **8**, **14,** and **18** were identified by matching with standards such as 5-*O*-caffeoylquinic acid (**2**), 3-*O*-caffeoylquinic acid (3CQA) (**6**), *trans*-caffeic acid (**8**), 1,5-dicaffeoylquinic acid (**14**), and 3,5-dicaffeoylquinic (**18**) in agreement with previous reports [13,28,34]. The total content of caffeoylquinic derivatives was 117.07 mg 3CQA equivalents/g PEE. 3CQA was the main compound in the PEE of *M. acuminata* with 90.03 mg/g PEE. High content of 3CQA also was reported in other polar extracts obtained from *M. friesiana* [35].

Compound **11** was identified by matching with commercial standards such as pinocembrin. Compounds **F_1_**, **F_2_**, **F_3_**, **F_4_**, **F_5_**, and **F_6_** were tentatively identified as flavanones. Compound **F_1_** presented UV spectra similar to the pinocembrin standard and the literature reports [30] suggesting that it could be a glycosylated pinocembrin derivative of the lower retention time. Compound **F_2_** presented a strong absorption of band II at 287 nm with a UV spectrum characteristic of tetrahydroxyflavanone derivatives [30]. Compounds **F_3_**, **F_4_**, **F_5_**, and **F_6_** presented λ_max_ at 282–287 with a characteristic shoulder at 310 nm in agreement with the UV spectra reported for trihydroxy methoxyflavanone derivative (**F_3_**), dihydroxy dimethoxyflavanone derivative (**F_4_**), and methoxyflavanone derivatives (**F_5_** and **F_6_**) [30]. The QTOF analysis of the PEE (Figure S1) shows signals in agreement with the tentative identification of **F_3_**, **F_4_**, **F_5_**, and **F_6_**. However, the presence of these compounds remains to be confirmed. Our results showed a total content of 144.01 mg NAR equivalents/g PEE. Compound **F_3_** was the main compound with 74.66 mg NAR equivalents/g PEE. In a study of different *Haplopappus* spp. (Asteraceae), high content of dihydroxy and trihydroxy dimethoxyflavanones was reported [36].

Compounds **15**, **17**, **20**, **23**, and **29** were identified by matching with standards such as quercetin-3-*O*-glucuronide (**15**), quercetin-3-*O*-rutinoside (**17**), quercetin-3-*O*-glucoside (**20**), isorhamnetin-3-*O*-rutinoside (**23**), and quercetin (**29**). The total flavonol content was 24.43 mg Q3G equivalents/g PEE. Compound **15** was the main flavonol with 17.80 mg Q3G equivalents/g PEE. Our results are in agreement with previous studies that reported a high content of quercetin-3-*O*-glucuronide in polar extracts from *M. acuminata* [11,13] and *M. friesiana* Cabrera [Asteraceae] [35]. Pelargonidin was detected in the extract, but at concentrations below the quantification limit.

#### 2.2.3. *Tagetes multiflora* Kunth [Asteraceae]

The HPLC-DAD chromatogram of the PEE of *T. multiflora* is shown in Figure 1c and the total content of compounds is shown in Table 1. Compounds **1** and **3** were identified as gallic acid (**1**) and vanillic acid (**3**) by matching with commercial standards. The total content of simple phenolic acids was 13.45 mg of *p*-HBA equivalents/g PEE. Vanillic acid was the major phenolic with 7.17 mg of *p*-HBA equivalents/g PEE Compounds **6** and **8** were identified by matching with standards such as 3-*O*-caffeoylquinic acid and *trans*-Caffeic acid, respectively. Compound **H_1_** was tentatively identified as caffeoylquinic acid derivative since it presented λ_max_ at 323 nm with a UV spectrum characteristic of coumaroylquinic acids derivatives [28]. The total content of HCAs was 11.74 mg 3CQA equivalents/g PEE. Our results are in agreement with the high content of gallic acid and caffeic acid that was found in fractions of methanolic extracts from *T. lucida* [37].

Flavonols were the main compounds in the PEE from *T. multiflora*. The presence of myricetin-3-*O*-glucoside (**12**), luteolin-7-*O*-glucoside (**13**), quercetin-3-*O*-rutinoside (**17**), quercetin-3-*O*-glucoside (**20**), quercetin-3-*O*-arabinoside (**21**), kaempferol-3-*O*-glucoside (**22**), quercetin-3-*O*-rhamnoside (**24**), quercetin (**29**), and isorhamnetin (**30**) was confirmed by matching with commercial standards. The total flavonol content was 276.90 mg Q3G equivalents/g PEE. In addition, compounds **F_1_**, **F_2_**, and **F_3_** presented λ_max_ 356–369 nm and UV spectra similar to quercetagetin derivatives according to the literature [30,38,39,40]. The compound **F_1_** was the main compound present in this species with 156.72 mg Q3G equivalents/g PEE. The QTOF analysis of the PEE of *T. multiflora* suggests the tentative identity of this compound as quercetagetin-hexoside derivative (Figure S2). In addition, Luteolin-7-*O*-glucoside, Kaempferol-3-*O*-glucoside, and Isorhamnetin were the other main flavonols with a content of 11.18, 8.00, and 8.08 mg naringenin equivalents/g PEE, respectively. Several studies have described a wide variety of glycosylated flavonoids present in the *Tagetes* species [41], but the high content of quercetagetin derivatives has been pointed out as a molecular marker of this genus [38,39,40]. Compound **F_4_** presented UV spectra similar to the luteolin standards and the literature reports [30]. Compounds **F_5_** and **F_6_** presented λ_max_ 347 nm and UV spectra similar to patuletin derivatives according to the literature [30]. Traces of pelargonidin were detected at concentrations below the quantification limit. So far, only the chemical composition of the essential oils from *T. multiflora* has been studied [19]. Abdala (2003) [42] studied the aerial parts of *T. gracilis* and reported flavonoids such as quercetagetin-7-*O*-glucoside, quercetagetin-3-*O*-glucoside, quercetagetin, quercetin-7-*O*-glucoside, quercetin-5-*O*-glucoside, quercetin-7-*O*-glucoside, luteolin-7-*O*-glucoside, and luteolin and suggested that this species is probably related to *T. multiflora* and could present similar type of flavonoids. However, to the best of our knowledge, no information can be found in the literature regarding the chemical characterization of polar extracts from *T. multiflora*, being this study the first report of the phenolic composition.

### 2.3. Cholinesterase Inhibitory Activities

Cholinesterase inhibitors are one of the most common approaches for the management of Alzheimer´s disease (AD) [24,43]. They can prevent the breakdown of acetylcholine (Ach), increasing the levels and improving the brain’s cognitive function [24,43]. However, most of them are linked with side effects [23,24,43]. Therefore, the search for new inhibitors from medicinal plants is an important task to find novel and safe medicines for AD [23,24,43]. Among many natural products that have effects on the AchE and BchE activities, polyphenols, phenolic acids, and stilbenes have been tested in vitro and ex vivo, showing inhibitory effects via a hydrogen bond, hydrophobic, and π- π mechanism of interaction [44]. In this study and given the diversity and content of phenolic compounds identified, the in vitro inhibitory effects of the PEE from *C. gilliesii*, *M. acuminata*, and *T. multiflora* was investigated.

Under our experimental conditions, the highest AChE inhibitory activity was obtained for *T. multiflora* (IC_50_ 298.17 ± 32.04 µg/mL) followed by *C. gilliesii* (IC_50_ 527.84 ± 32.00 µg/mL), while the PEE of *M. acuminata* was not active against AChE (IC_50_ > 1000 µg/mL). In the BChE inhibition assays, all PEE inhibited the enzyme activity. *T. multiflora* showed an IC_50_ value of 583.52 ± 25.53 µg/mL, while *M. acuminata* presented an IC_50_ of 668.15 ± 39.07 µg/mL, and *C. gilliesii* an IC_50_ of 725.209 ± 27.62 µg/mL. The reference compound Galantamine showed IC_50_ values of 0.57 ± 0.05 µg/mL for AChE and 8.08 ± 0.02 µg/mL for BChE. A simple trend can be observed regarding the total content of polyphenols and the inhibitory activity of the extracts against both enzymes, in agreement with previous studies that reported a correlation between flavonoids and acetylcholinesterase inhibitory activity [20]. Compared with results in the literature among other Chilean Altiplano plants, the observed effects herein can be considered weak. The cholinesterase inhibitory activity of *Artemisia copa* Phil. (Asteraceae) was evaluated, showing IC_50_ values of 3.92 ± 0.08 µg/mL and 44.13 ± 0.10 µg/mL) against AChE and BChE, respectively [24].

The cholinesterase inhibitory activity of some *Clinopodium* species has been investigated previously. The ethanolic extract of the Argentinian *C. gilliesi* showed inhibitory activities against AChE (IC_50_ 1360.0 ± 75.9 µg/mL) and BChE (IC_50_ 1645.9 ± 51.2 µg/mL) [15]. A selective BChE inhibitory activity was reported for the essential oils from Ecuadorian *Clinopodium taxifolium* (Kunth) [45] and *Clinopodium brownie* (Sw.) Kuntze [Lamiaceae] [46], yielding IC_50_ values of 31.3 ± 3.0 µg/mL and 13.4 ± 1.8 µg/mL, respectively. Recently, the methanol and hot-water extracts from *Clinopodium vulgare* L. [Lamiaceae] showed less than 15% inhibitory activity at 1000 µg/mL [47]. In another study, the acetone, methanol, and water extracts of *Clinopodium vulgare* spp showed 1.34 ± 0.01, 1.08 ± 0.03, and 0.25 ± 0.01 mg galantamine equivalents/g of the extract against AChE, while against BChE, only the acetone extract was reported as active, with 0.93 ± 0.23 mg galantamine equivalents/g of extract. [48]. From the Algerian plant *Clinopodium nepeta* (L.) Kuntze [Lamiaceae], only the dichloromethane extract showed IC_50_ values against AChE (170.1 ± 1.58 µg/mL), while the ethyl acetate and dichloromethane extracts were inhibitors of BChE, with IC_50_ values of 187.8 ± 1.57 and 73.06 ± 0.83 µg/mL, respectively [49]. Among *Tagetes* genus, the ethanolic extract obtained from orange and yellow flowers of *T. erecta* was investigated against AChE, showing IC_50_ values > 1000 µg/mL for both cultivars [50]. No investigations have been reported regarding cholinesterase inhibition for species of the *Mutisia* genus. To the best of our knowledge, this is the first report concerning the inhibitory capacity of *C. gilliesii*, *M. acuminata*, and *T. multiflora* species against these enzymes.

### 2.4. Molecular Docking

Although our results of IC_50_ calculated for the PEE from *C. gilliesii*, *M. acuminata*, and *T. multiflora* are higher than that achieved by galantamine (reference drug), the PEE can be considered active, given that the extracts are complex mixtures of compounds, including structures that can be inactive. For a better understanding of the effects observed, and to study if the main individual compounds present in these species could have effects on AChE and BChE, an *in silico* study was carried out. Since the PEE from *T. multiflora* presented the best AChE and BChE inhibitory activities, the interactions of AChE and BChE with the main compounds present in this extract were evaluated. The docking analysis showed that all the compounds analyzed presented hydrogen bonding (HB) interactions within the active site of both AChE and BChE enzymes. Compounds present in their structure hydroxyl groups can generate this interaction with side chains or backbones of some residues. In BChE, six of the analyzed compounds presented HB with the Gly115 and Tyr128 residues, located in the oxyanion hole and anionic site, respectively.

On the other hand, the tested compounds showed interactions with residues located in the peripherical anionic site of AChE, around the entrance to the active site cavity. About 86% of the studied compounds showed HB with the Phe295 residue, followed by Asp74, which interacted with four compounds. Unlike BChE, the ligand–AChE complexes exhibited π-π stacking-type interactions with residues such as Trp286, Tyr341, His447, and Phe338 (Table 2).

**Figure 2:**
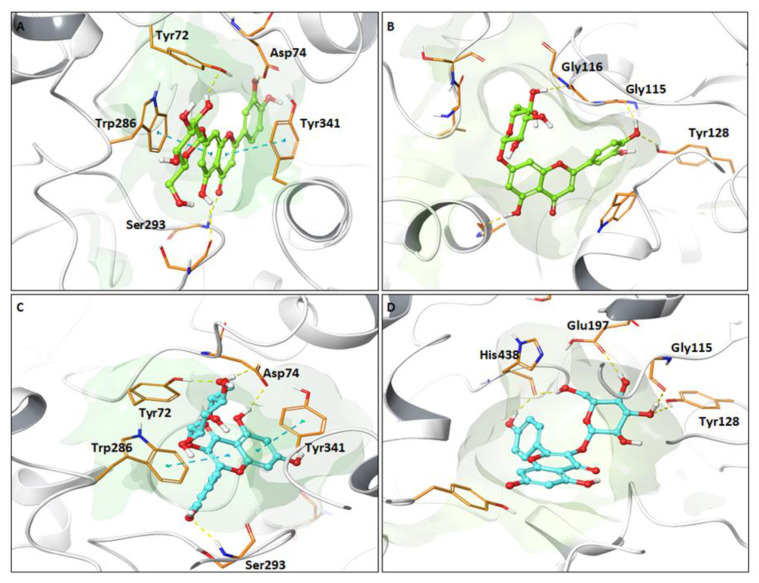
Predicted binding modes for some studied compounds within the AChE and BChE active sites. (**A**) Luteolin-7-*O*-glucoside is shown in ball-and-stick representation with green carbons within AChE and (**B**) BChE active site. (**C**) Kaempferol-3-*O*-glucoside is shown in ball-and-stick representation with cyan carbons within AChE and (**D**) BChE active site. Relevant amino acids are shown in the tubes in orange. The secondary protein structure is depicted as white ribbons. Cyan dotted lines represent π-π stacking interaction between ligands and AChE/BChE residues. Yellow dotted lines represent hydrogen bond interaction between ligands and AChE/BChE residues.

Figure 2 shows the binding mode of luteolin-7-*O*-glucoside (**13**) and kaempferol-3-*O*-glucoside (**22**) with AChE and BChE, respectively. Both compounds present π-π stacking interactions with residues Trp286 and Tyr341 located on the periphery of the active site. In addition, the two compounds show HB contacts with the amino acids Tyr72, Asp74, and Ser293. It should be noted that the interaction with Asp74 is generated by a glycosidic hydroxyl.

In the same way, compounds **13** and **22** shared similar interactions in the BChE active site, as HB with residues Tyr128 and Gly115. The binding mode of the rest of the compounds is shown in Figure S3. Compared to other studies, Kuppusamy highlighted that molecular docking of several flavonoids confirms the presence of HB and pi stacking interactions in the AChE binding site with residues such as Tyr124, Trp286, Ser293, Phe295, and Tyr341, which coincide with the present study [51]. Khan et al. [52] described the interactions of quercetin in the active site of cholinergic enzymes, highlighting hydrogen bond contacts with Asp74 in AChE and Tyr128 within the active site of BChE. The lack of π-π stacking interaction in BChE is because the residues of this active site are mainly aliphatic, while AChE has several amino acids with aromatic side chains [53]. Likewise, these additional interactions can make ligand–AChE complexes more stable than BChE complexes, consistent with the calculated binding energy, evidenced in Table 3.

## 3. Materials and Methods

### 3.1. Standards and Reagents

From Sigma-Aldrich (St. Louis, MO, USA): Amberlite^®^ XAD7HP, gallic acid, vanillic acid, salicylic acid, 5-*O*-caffeoylquinic acid, pinocembrin, dimethyl sulfoxide (DMSO), and acetylcholinesterase (AChE) from *Electrophorus electricus* (electric eel), and butyrylcholinesterase (BChE) from equine serum. From PhytoLab (Vestenbergsgreuth, Germany): *p*-hydroxybenzoic acid, 3-*O*-caffeoylquinic acid, *trans*-caffeic acid, 1,5-dicaffeoylquinic acid, 3,5-dicaffeoylquinic acid, (*+*)-catechin, B-type procyanidin dimer ((*epi*)catechin-(*epi*)catechin), naringin, naringenin, luteolin-7-*O*-glucuronide, myricetin-3-*O*-glucoside, luteolin-7-*O*-glucoside, quercetin-3-*O*-glucuronide, quercetin-3-*O*-galactoside, quercetin-3-*O*-rutinoside, quercetin-3-*O*-glucoside, quercetin-3-*O*-arabinoside, kaempferol-3-*O*-glucoside, isorhamnetin-3-*O*-rutinoside, quercetin-3-*O*-rhamnoside, isorhamnetin-3-*O*-glucoside, apigenin-7-*O*-glucoside, luteolin, quercetin, isorhamnetin, and pelargonidin chloride. From Merck (Darmstadt, Germany): HPLC-grade methanol, acetonitrile, formic acid, and ethanol. Ultrapure water was obtained using a Barnstead EasyPure water filter (Thermo Scientific, Waltham, MA, USA).

### 3.2. Plant Material and Preparation of Extracts

The aerial parts from *C. gilliesii* (Muña-Muña), *M. acuminata* (Chinchircoma), and *T. multiflora* (Gracilis) were collected in March of 2019 in Putre, Región de Arica y Parinacota, Chile. The samples were identified and deposited in our laboratory herbarium (voucher numbers: Cg-032019, Ma-032019 and Tm-032019). The samples were cleaned with water and dried in an oven at 35 °C. A total of 10 g of dried aerial parts for each species were ground and extracted with 100 mL of EtOH using an ultrasonic water bath (UC-60A Biobase, Guanzhou, China). To clean-up crude extracts and to make polyphenolic enriched-extracts (PEEs), an Amberlite^®^ XAD7HP resin was used. The column was washed with water and compounds were desorbed with MeOH. The PEEs were evaporated under reduced pressure and freeze dried for further analyses.

### 3.3. HPLC-DAD Analysis and Quantification

The HPLC-DAD system used was Shimadzu equipment (Shimadzu Corporation, Kyoto, Japan) consisting of an LC-20AT pump, an SPD-M20A UV diode array detector, a CTO-20AC column oven, and LabSolution software. A MultoHigh^®^ RP18 (250 × 4.6 mm, 5 µm of particle size) column (CS-Chromatographie Service GmbH, Langerwehe, Germany). The column temperature was set at 30 °C. The HPLC analyses were performed using a linear gradient solvent system consisting of H_2_O-formic acid-ACN (88.5:8.5:3, *v*/*v*/*v*, solvent A), H_2_O-formic acid-ACN (41.5:8.5:50, *v*/*v*/*v*, solvent B), and MeOH-formic acid-ACN (90:8.5:1.5, *v*/*v*/*v*, solvent C) with a flow rate of 0.4 mL/min as follows: the initial conditions were 98%A, 2%B, and 0%C; at min 22, 70%A, 17%B, and 13%C; at min 52, 60%A, 25%B, and 15%C; at min 30, 60%A, 40%B, and 30%C; at min 75, 0%A, 50%B, and 50%C; and at min 85, 98%A, 2%B, and 0%C. The column was stabilized for an additional 10 min in the same gradient (98%A:2%B:0%C) before the next injection. The injected volume of the PEEs from each species was 20 µL at a concentration of 5 mg/mL. The compounds were monitored at 280, 320, 354, and 520 nm, and UV spectra from 200 to 600 nm were recorded for peak characterization.

The compounds in each PEEs were identified by matching retention times and UV–vis spectra with commercial standards and literature related to the species in this study. The UV absorbance profile of compounds without matching was used for the tentative identification of groups of compounds. Each peak UV profile was assessed and compounds were classified and quantified by the typical absorption band I of the B-ring cinnamoyl structure and band II of the A-ring benzoyl or benzene structure [54,55], as follows: simple phenols derivatives, flavan-3-ol monomers and polymers, and flavanones were identified by the absorption band II with λ_max_ at 280 nm; hydroxycinnamic acid derivatives were identified by the absorption band I with λ_max_ at 320–329 nm; flavonol derivatives were identified by the absorption band I with λ_max_ at 354–370 nm; and anthocyanin compounds were identified by the strong visible absorption band at 500–520 nm. Quantification of total simple phenols derivatives (S), hydroxycinnamic acids (H), and flavonoids (F), such as flavan-3-ol monomers and polymers, flavanones, flavonols, and anthocyanins was carried out using external calibration curves with commercial standards of *p*-hydroxybenzoic acid (*p*-HBA), 3-*O*-caffeoylquinic acid (3CQA), (*+*)-catechin (CAT), naringenin (NAR), quercetin-3-*O*-glucoside (Q3G), and pelargonidin chloride (PhytoLab, Vestenbergsgreuth, Germany). All concentrations are expressed as mg of the corresponding compound/g of PEE. The analytical parameters were in agreement with the International Conference on Harmonization as follows: for simple phenols derivatives limit of detection (LOD): 5.58 µg/mL, the limit of quantification (LOQ): 16.91 µg/mL; for hydroxycinnamic acids LOD: 8.67 µg/mL, LOQ: 26.28 µg/mL; Flavan-3-ol monomers and polymers LOD: 2.53 µg/mL, LOQ: 7.67 µg/mL; flavanones LOD: 1.12 µg/mL, LOQ: 3.39 µg/mL; for flavonols: LOD: 2.71 µg/mL, LOQ: 8.21 µg/mL; and for anthocyanins: LOD: 0.13 µg/mL; LOQ: 0.41 µg/mL. In addition, the main compounds present in PEEs from *M. acuminata* and *T. multiflora* were tentatively identified by ESI-MS-MS high-resolution analysis as previously reported [56] (Methodology S1: High-Resolution Mass Spectrometry in the Supplementary Materials section). The chemical composition of extracts complies with the ConPhyMP statement [57].

### 3.4. Determination of Cholinesterase Inhibition

The in vitro inhibitory potential of PEE from *C. gilliesii, M. acuminata*, and *T. multiflora* were evaluated by Ellman’s method. PEE of each sample (50 µL, 2 mg/mL) was mixed with 120 µL of 5,5-dithio-bis (2-nitrobenzoic) acid (DTNB) 0.3 mM, AChE (0.26 U/mL, from Electric eel), or BChE (0.26 U/mL, from horse serum) solution (25 µL) in Tris-HCl buffer 50 mM (pH = 8.0) in a 96-well microplate and incubated for 20 min at 37 °C. The reaction was initiated by the addition of 25 µL of acetylthiocholine iodide (ATCI) 1.5 mM or butyrylthiocholine chloride (BTCl) 1.5 mM. The absorbances were recorded three times at 405 nm at 37 °C using a microplate reader (Multiskan EX, Thermo). Galantamine hydrobromide was used as a positive control. The cholinesterase inhibitory activity was expressed as IC_50_ (µg mL^−1^, concentration range 15 to 500 µg mL^−1^). All data were recorded in triplicate.

### 3.5. Molecular Docking

The crystallographic structures of AChE in complex with donepezil (PDB ID: 4EY7, Resolution: 2.3 Å) [58] and BChE in complex with tacrine (PDB ID: 4BDS, Resolution: 2.1 Å) [59] were obtained from the Protein Data Bank (PDB) (http://www.rcsb.org (accessed on 28 October 2022)). These protein structures were prepared for molecular docking using the *Preparation Wizard* module from suite Maestro [60]. The three-dimensional structures of the compounds were downloaded from PubChem (https://pubchem.ncbi.nlm.nih.gov (accessed on 28 October 2022)) and prepared through the *Ligprep* module [60] under the OPLS-AA force field [13] to generate the minimized energy conformations. Molecular docking studies were performed using *Glide* from suite Maestro. *Glide* uses a series of hierarchical filters to find the best ligand binding posse in a protein grid space [61]. The original co-crystallized ligands’ location was used as the grid box center, which was refined and set up to 20 Å. Initially, the ligands were docked using the standard precision algorithm (SP), and the best-ranked poses were re-docked with the extra precision algorithm (XP). The glide score, interactions, and visually inspected were used as a criterion for selecting the best poses.

### 3.6. Statistical Analysis

The results are expressed as mean values ± standard error using GraphPad Prism 8 (GraphPad Software Corporation, La Jolla, CA, USA). One-way analysis of variance (ANOVA; repeating three times each measurement of sample solutions) (*p* < 0.05) was used for the comparison of means.

## 4. Conclusions

This is the first report on the identification and characterization of phenolic compounds from three Chilean Altiplano plants: *Clinopodium gilliesii*, *Mutisia acuminata*, and *Tagetes multiflora*. In this study, 30 phenolic compounds were tentatively identified, including simple phenolics, hydroxycinnamic acids, flavan-3-ols (monomers and polymers), flavanones, and flavonols. The three PEEs showed mild inhibitory activity against cholinesterase enzymes, with higher IC_50_ values achieved by *T. multiflora* extract. Docking analysis for main selected compounds tentatively identified in *T. multiflora,* including gallic acid, vanillic acid, luteolin-7-*O*-glucoside, quercetin-3-*O*-arabinoside, quercetin, isorhamnetin, and kaempferol-3-*O*-glucoside, indicated that hydrogen bonding interaction and π-π stacking-type interactions were predominant in the experiments, partially explaining the observed effects. The main individual compounds inhibited the AD-associated enzymes (AChE and BChE) through π-π stacking interactions with Trp286 and Tyr341 residues located on the periphery of the active site in both enzymes. This is the first evidence about the potential neuroprotective effect of phenolic compounds from *C. gilliesii*, *M. acuminata*, and *T. multiflora*.

## Figures and Tables

**Table 1:** Tentative identification and content of phenolic compounds from PEEs of *C. gilliesii*, *M. acuminata*, and *T. multiflora*.

Peak	Rt (min)	λmax (nm)	Compound Identification	*C. gilliesii*	*M. acuminata*	*T. multiflora*
**Simple phenolics**						
**1**	9.89	272, 245sh, 235	Gallic acid	ND	ND	6.28 ± 0.17
**3**	12.65	260, 293, 231	Vanillic acid	12.37 ± 0.36	ND	7.17 ± 0.32
**4**	17.74	257, 233	*p*-Hydroxybenzoic acid	1.43 ± 0.09	ND	ND
**10**	29.63	301, 245, 233	Salicylic acid	ND	15.18 ± 0.03	ND
			***Total content* ^1^**	**13.80 ± 0.45**	**66.99 ± 2.29**	**13.45 ± 0.49**
**Hydroxycinnamic acids**						
**2**	12.22	324, 300sh, 245	5-*O*-Caffeoylquinic acid	ND	5.92 ± 0.01	ND
**6**	23.19	325, 300sh, 240	3-*O*-Caffeoylquinic acid	1.14 ± 0.04	90.03 ± 0.46	2.17 ± 0.01
**8**	25.91	323, 300sh, 265, 240	*trans*-Caffeic acid	1.56 ± 0.10	2.74 ± 0.01	2.05 ± 0.06
**14**	43.21	323, 300sh, 255, 233	1,5-Dicaffeoylquinic acid	ND	9.86 ± 0.02	ND
**18**	45.83	327, 395sh, 257, 233	3,5-Dicaffeoylquinic acid	ND	8.51 ± 0.01	ND
			***Total content* ^2^**	**4.27 ± 0.35**	**117.07 ± 0.50**	**11.74 ± 0.27**
**Flavan-3-ol monomers and polymers**						
**5**	21.67	282, 249, 231	(+)-Catechin	0.40 ± 0.00	ND	ND
**7**	24.10	279, 249, 230	B-type Procyanidin dimer-(epi)Catechin-(epi)Catechin	5.75 ± 1.06	ND	ND
			***Total content* ^3^**	**70.00 ± 4.87**	**0.00 ± 0.00**	**0.00 ± 0.00**
**Flavanones**						
**11**	33.11	293, 330sh, 250	Pinocembrin	ND	1.98 ± 0.01	ND
**19**	46.86	283, 330sh, 250	Naringin	1.67 ± 0.01	ND	ND
**26**	65.07	289, 330sh, 250	Naringenin	0.09 ± 0.00	ND	ND
			***Total content* ^4^**	**93.08 ± 2.75**	**144.01 ± 1.25**	**0.00 ± 0.00**
**Flavonols**						
**9**	29.33	348, 265sh, 255, 236	Luteolin-7-*O*-glucuronide	1.33 ± 0.01	ND	ND
**12**	39.06	344, 273, 248	Myricetin-3-*O*-glucoside	ND	ND	1.35 ± 0.00
**13**	42.43	348, 267sh, 245, 236	Luteolin-7-*O*-glucoside	15.72 ± 0.17	ND	11.18 ± 0.46
**15**	43.84	353, 258sh, 230	Quercetin-3-*O*-glucuronide	ND	17.80 ± 0.20	ND
**16**	44.52	354, 270sh, 250	Quercetin-3-*O*-galactoside	1.53 ± 0.14	ND	ND
**17**	45.10	354, 263sh, 230	Quercetin-3-*O*-rutinoside	ND	2.49 ± 0.08	3.36 ± 0.11
**20**	49.90	354, 268sh, 256	Quercetin-3-*O*-glucoside	1.63 ± 0.07	1.05 ± 0.06	3.93 ± 0.83
**21**	51.86	353, 257, 237	Quercetin-3-*O*-arabinoside	ND	ND	6.07 ± 1.15
**22**	56.40	364, 263, 231	Kaempferol-3-*O*-glucoside	1.20 ± 0.14	ND	8.00 ± 0.89
**23**	62.30	365, 290, 231	Isorhamnetin-3-*O*-rutinoside	0.45 ± 0.00	1.21 ± 0.00	ND
**24**	62.68	348, 257, 238	Quercetin-3-*O*-rhamnoside	ND	ND	2.27 ± 0.53
**25**	64.50	365, 268, 250	Isorhamnetin-3-*O*-glucoside	0.10 ± 0.00	ND	ND
**27**	67.40	337, 267, 244	Apigenin-7-*O*-glucoside	1.07 ± 0.05	ND	ND
**28**	69.80	347, 296sh, 267, 256	Luteolin	3.33 ± 0.25	ND	ND
**29**	71.82	369, 249, 256	Quercetin	ND	1.89 ± 0.19	5.29 ± 0.20
**30**	74.59	370, 268, 231	Isorhamnetin	ND	ND	8.08 ± 0.15
			***Total content* ^5^**	**48.39 ± 2.25**	**24.43 ± 0.54**	**276.90 ± 7.2**

Rt: retention time. ND: not detected. Superscript numbers (1–5) indicate the total content (including compounds not identified by standards) expressed as **^1^** total simple phenolics content is expressed as mg *p*-hydroxybenzoic acid equivalents/g PEE; **^2^** total hydroxycinnamic acids content is expressed as mg 3-*O*-caffeoylquinic acid equivalents/g PEE; **^3^** total flavan-3-ol monomers and polymers content is expressed as mg (+)-catechin equivalents/g PEE; **^4^** total flavanones content is expressed as mg naringenin equivalents/g PEE; **^5^** total flavonols content is expressed as mg quercetin-3-*O*-glucoside equivalents/g PEE.

**Table 2:** Specific AChE/BChE–ligand interactions and their occurrence (%) and averaged distances, obtained from molecular docking experiments.

	AChE			BChE		
Interaction Type	Residues	N ^a^	Distance (Å)	Residues	N ^a^	Distance (Å)
**H-Bond**	Phe295	6 (86%)	1.94 ± 0.11	Gly115	6 (86%)	1.87 ± 0.27
	Asp74	4 (57%)	2.00 ± 0.22	Tyr128	6 (86%)	2.14 ± 0.21
	Ser293	3 (43%)	2.53 ± 0.31	His438	2 (29%)	1.82 ± 0.06
	Tyr72	2 (29%)	1.90 ± 0.33	Ala328	2 (29%)	2.25 ± 0.49
	Gly122	2 (29%)	2.26 ± 0.07	Tyr332	2 (29%)	2.01 ± 0.21
	Ser203	2 (29%)	1.77 ± 0.01	Glu197	1 (14%)	2.14 ± 0.00
	His287	1 (14%)	2.13 ± 0.00	Gly116	1 (14%)	2.19 ± 0.00
**π-stacking**	Tyr341	4 (57%)	3.84 ± 0.19			
	Trp286	3 (43%)	4.38 ± 0.88			
	His447	2 (29%)	4.96 ± 0.02			
	Phe338	2 (29%)	4.75 ± 0.01			

^a^ represents the number of compounds that interact with a specific residue.

**Table 3:** Binding energy of enzyme–ligand complexes.

Compounds	Binding Energy(kcal/mol) *	
	AChE	BChE
Luteolin-7-*O*-glucoside (**13**)	−13.471	−12.380
Quercetin-3-*O*-arabinoside (**21**)	−11.728	−9.950
Quercetin (**29**)	−11.359	−9.863
Isorhamnetin (**30**)	−9.980	−8.742
Kaempferol-3-*O*-glucoside (**22**)	−9.949	−8.973
Gallic acid (**1**)	−5.467	−6.085
Vanillic acid (**3**)	−5.060	−5.543

* empirical scoring function that approximates the ligand binding free energy. As it simulates binding free energy, more negative values represent tighter binders.

## Data Availability

All the data are contained within the article.

## Supplementary Materials

The following supporting information can be downloaded at: https://www.mdpi.com/article/10.3390/plants12040819/s1, Methodology S1: High-Resolution Mass Spectrometry; Figure S1: High-resolution Mass Spectrometry analysis of the main compounds present in PEE from the aerial parts of *Mutisia acuminata*; Figure S2: High-resolution Mass Spectrometry analysis of the main compounds present in PEE from the aerial parts of *Tagetes multiflora*; Figure S3: Predicted binding modes for some studied compounds within the AChE and BChE active sites.
