# Supplementary material for: Phenolic Profile and Cholinesterase Inhibitory Properties of Three Chilean Altiplano Plants: Clinopodium gilliesii (Benth.) Kuntze [Lamiaceae], Mutisia acuminata Ruiz & Pav. var. hirsuta (Meyen) Cabrera, and Tagetes multiflora (Kunth) [Asteraceae]
Source: Plants (Basel). 2023 Feb 12;12(4):819. doi: 10.3390/plants12040819 (PMC9960489; doi:10.3390/plants12040819)
Supplement: Supplementary file 1 [file plants-12-00819-s001.zip › plants-2190448-supplementary.pdf]

plants

MDPI

Article

Phenolic Profile and Cholinesterase Inhibitory Properties of Three Chilean Altiplano Plants: *Clinopodium gilliesii* (Benth.) Kuntze [Lamiaceae], *Mutisia acuminata* Ruiz & Pav. var. hirsuta (Meyen) Cabrera, and *Tagetes multiflora* (Kunth) [Asteraceae]

Supplementary Materials

Methodology S1: *High-Resolution Mass Spectrometry*

The main compounds present in PEEs from *Mutisia acuminata* and *Tagetes multiflora* were tentatively identified by spectrometric means. ESI-MS-MS High-Resolution analyses were conducted in a Micromass Q-TOF micro instrument (Manchester, UK), according to Mieres-Castro et al. (2019). Briefly, the PEEs were directly infused at a flow rate of 10.0 μL/min using a syringe pump (Harvard Apparatus, Holliston, MA, USA). ESI mass and tandem mass spectra were acquired in the negative ion mode. The following operating conditions were used: 3.0 kV capillary voltage, 40V cone voltage, and 100°C for de-solvation gas. Tandem ESI-MS-MS spectra were collected by collision-induced dissociation (CID) of the mass-selected molecules, using argon as the buffer gas and 5-45 eV of collision energies. The mass selection was performed by quadrupole 1 using a unitary *m*/*z* window, and collisions were performed in the rf-only quadrupole collision cell, followed by time-of-flight (TOF) mass analysis. The spectra were recorded over an m/z range of 50-700 amu.

Mieres-Castro, D.; Schmeda-Hirschmann, G.; Theoduloz, C.; Gómez-Alonso, S.; Pérez Navarro, J.; Márquez, K.; Jiménez-Aspee, F. Antioxidant activity and isolation of polyphenols and new iridoids from Chilean Gaultheria phillyreifolia and G. poeppigii berries. Food Chem. 2019, 291, 167–179. doi: 10.1016/j.foodchem.2019.04.019

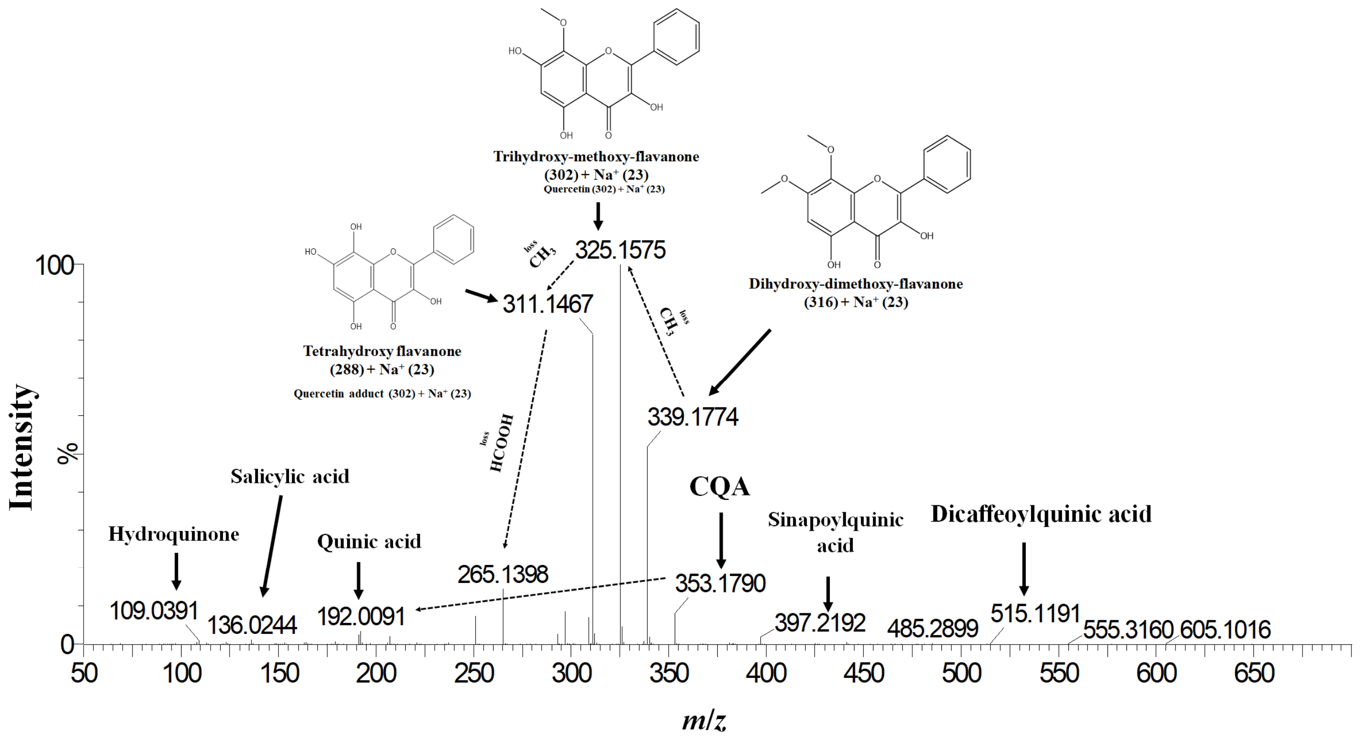

Figure S1. High-resolution Mass Spectrometry analysis of the main compounds present in PEE from the aerial parts of *Mutisia acuminata*.

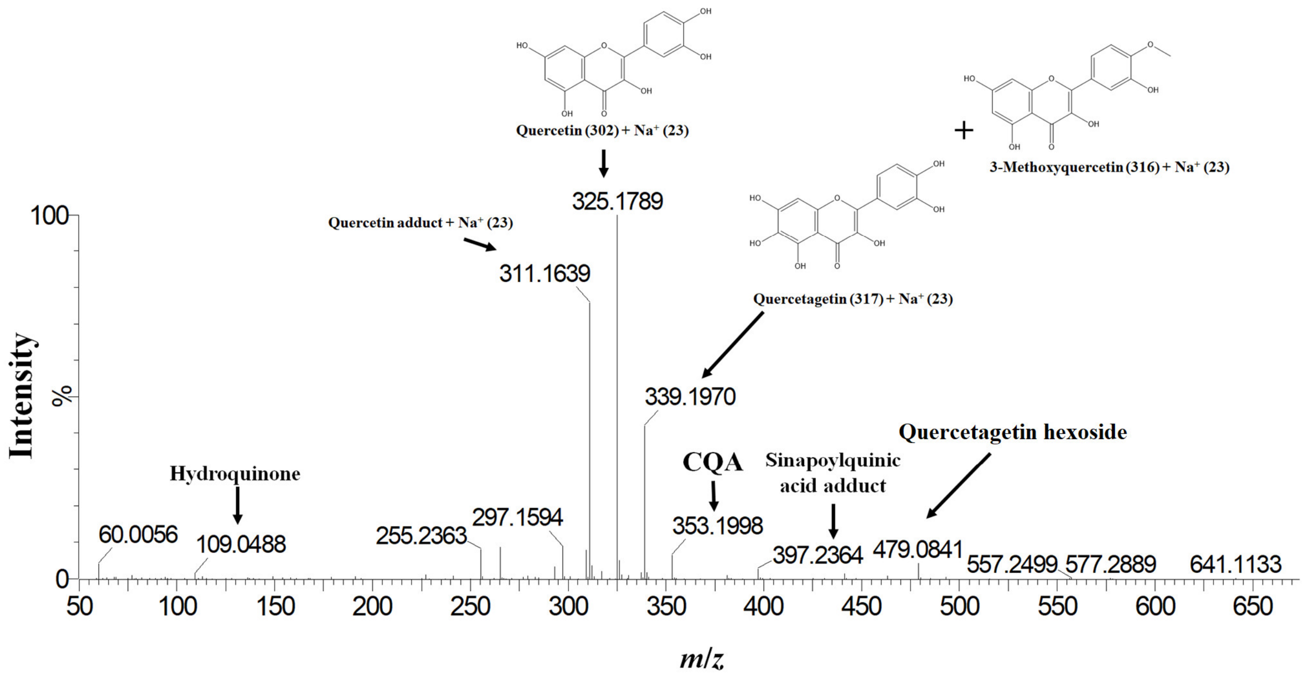

Figure S2. High-resolution Mass Spectrometry analysis of the main compounds present in PEE from the aerial parts of *Tagetes multiflora*.

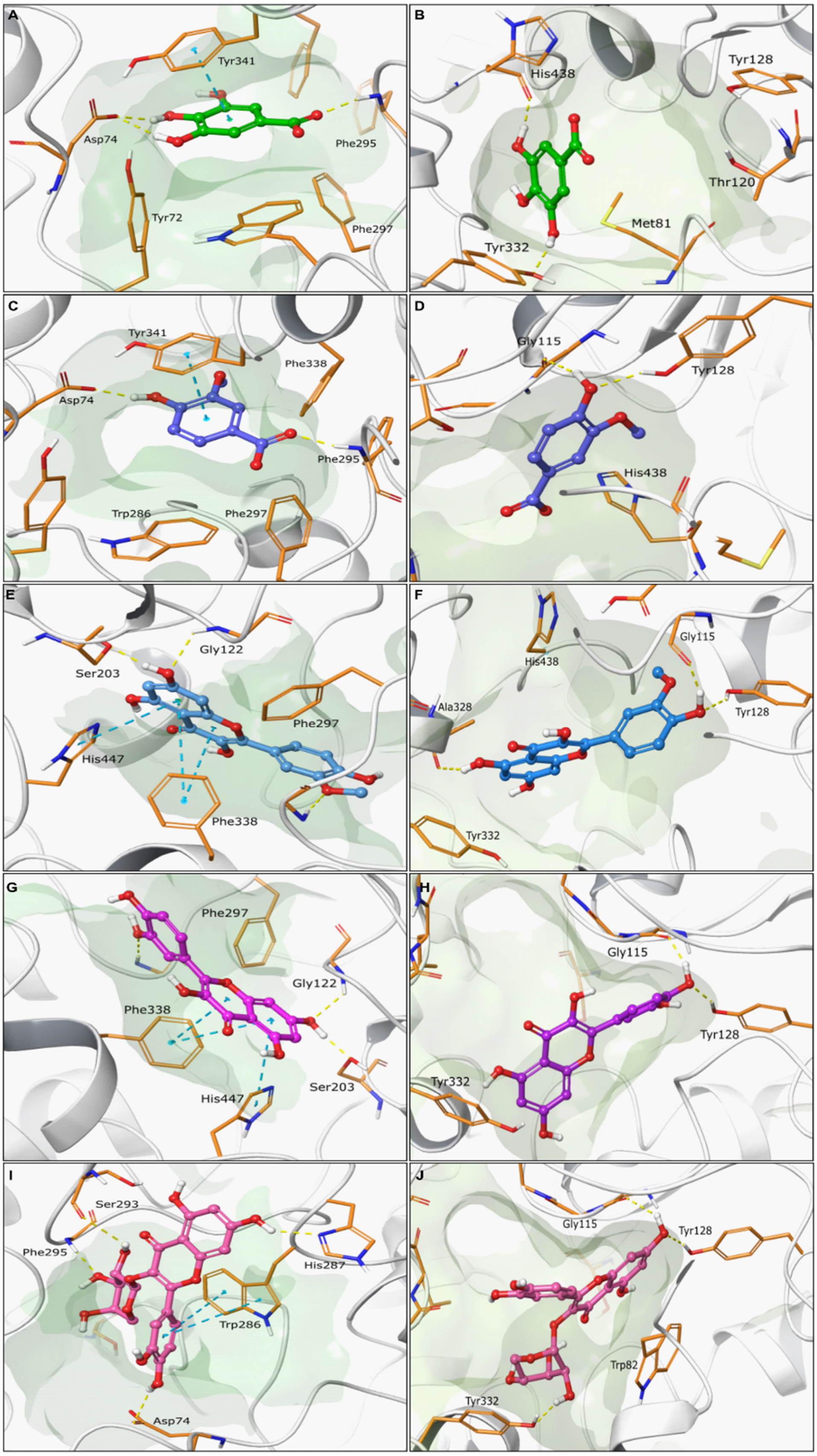

Figure S3. Predicted binding modes for some studied compounds within the AChE and BChE active sites. (A) Gallic acid (green carbons) within AChE and (B) BChE active site. (C) Vanillic acid (violet carbons) within AChE and (D) BChE active site. (E)

Isorhamnetin (faded azure carbons) within AChE and (F) BChE active site. (G) Quercetin (magenta carbons) within AChE and (H) BChE active. (I) Quercetin-3-*O*-arabinoside (pink carbons) within AChE and (J) BChE active site. Ligands are shown in ball-and-stick representation. Relevant amino acids are shown in the tubes in orange. The secondary protein structure is depicted as white ribbons. Cyan dotted lines represent π-π stacking interaction between ligands and AChE/BChE residues. Yellow dotted lines represent hydrogen bond interaction between ligands and AChE/BChE residues.
